# Supplementary material for: Reconciling Mining with the Conservation of Cave Biodiversity: A Quantitative Baseline to Help Establish Conservation Priorities
Source: PLoS One. 2016 Dec 20;11(12):e0168348. doi: 10.1371/journal.pone.0168348 (PMC5173368; doi:10.1371/journal.pone.0168348)
Supplement: S1 Dataset — (ZIP) [file pone.0168348.s002.zip › Taxa/Serra Norte/SN_2013/N5S/Lista N5S-74.pdf]

CAVIDADE N5S-0074

Classe	Ordem	Fam/Outros	Gên/Outros	Espécie	S-U	U-S	Zon.
Annelida	Oligochaeta			jovem	2	5	P/A
Arachnida	Acari	Ascidae		sp.1		X	E/P
Arachnida	Acari	Eupodidae		sp.1	X	X	E/P/A
Arachnida	Acari	Eutrombidiidae		sp.66	X		A
Arachnida	Acari	Ixodidae	*Ixodes*	sp.1		X	A
Arachnida	Acari	Laelapidae		sp.3		X	A
Arachnida	Acari	Mesostigmata		jovem	X		A
Arachnida	Acari	Neothyridae	*Diplothyrus*	sp.1		X	E/P/A
Arachnida	Acari	Rhagidiidae		sp.20	X		P
Arachnida	Acari	Trombidiformes		sp.49	X		A
Arachnida	Amblypygi	Charinidae	*Charinus*	sp.1	4	8	A
Arachnida	Amblypygi	Phrynidae	*Heterophrynus*	*longicornis*	6	5	P/A
Arachnida	Araneae	Caponiidae		sp.1	X	X	P/A
Arachnida	Araneae	Corinnidae	*Creugas*	sp.1	3	8	P/A
Arachnida	Araneae	Ctenidae		jovem	7	1	E/P/A
Arachnida	Araneae	Ochyroceratidae	*Ochyrocera*	sp.3		X	P
Arachnida	Araneae	Ochyroceratidae	*Speocera*	sp.1	X	X	P/A
Arachnida	Araneae	Oonopidae		jovem		X	P
Arachnida	Araneae	Pholcidae	*Mesabolivar*	*aurantiacus*		X	
Arachnida	Araneae	Pholcidae	*Mesabolivar*	sp.1	X	X	E/P
Arachnida	Araneae	Pisauridae	*Architis*	sp.1	2	2	E/P
Arachnida	Araneae	Prodidomidae	*Lygromma*	sp.1		X	A
Arachnida	Araneae	Theraphosidae	aff. *Holothele*	sp.2		1	
Arachnida	Araneae	Theridiosomatidae	*Plato*	sp.1	X	X	E/P
Arachnida	Opiliones	Escadabiidae		sp.1	X	X	P/A
Arachnida	Opiliones	Sclerosomatidae	*Prionostema*	sp.1		3	
Arachnida	Palpigradi	Eukoeneniidae	*Allokoenenia*	sp.1	X	X	P/A
Arachnida	Palpigradi	Eukoeneniidae	*Eukoenenia*	sp.1		X	A
Arachnida	Pseudoscorpiones	Chthoniidae		jovem		X	A
Arachnida	Ricinulei	Ricinoididae	*Cryptocellus*	*tarsilae*	1		A
Chilopoda	Geophilomorpha	Geophilidae		sp.1		1	P
Chilopoda	Scolopendromorpha	Scolopendridae	*Otostigmus*	sp.1		1	A
Chilopoda	Scolopendromorpha	Scolopocryptopidae	*Dinocryptops*	*miersii*		1	A
Chilopoda	Scolopendromorpha	Scolopocryptopidae	*Newportia*	sp.1		2	P/A
Chilopoda	Scutigeromorpha	Pselliodidae	*Sphendononema*	jovem	2		P
Diplopoda	Polydesmida	Chelodesmidae		jovem	1	1	E/P/A
Diplopoda	Polydesmida	Fuhrmannodesmidae		jovem	X	X	P/A
Diplopoda	Polydesmida	Pyrgodesmidae		sp.1	X	X	P/A
Entognatha	Collembola	Cyphoderidae		sp.1		X	A
Entognatha	Collembola	Entomobryidae		sp.4		X	P
Entognatha	Collembola	Isotomidae		sp.1	X	X	E/P/A
Entognatha	Collembola	Paronellidae		sp.1	X	X	E/P/A
Entognatha	Collembola	Paronellidae		sp.7		X	P
Entognatha	Collembola	Sminthuroidea		sp.1		X	A
Entognatha	Collembola	Sminthuroidea		sp.2	X		P/A
Entognatha	Diplura	Campodeidae		sp.1		X	E/P
Insecta	Blattodea	Blattellidae		sp.1		1	P
Insecta	Blattodea	Blattellidae		sp.5		1	
Insecta	Blattodea			jovem	X		A

Insecta	Coleoptera	Carabidae		sp.3		X	E/P/A
Insecta	Coleoptera	Curculionidae	Scolytinae	sp.1	X		A
Insecta	Coleoptera	Dytiscidae		sp.1		X	P
Insecta	Coleoptera	Dytiscidae		sp.5	X		P
Insecta	Coleoptera	Dytiscidae		sp.12B		X	P
Insecta	Coleoptera	Scydmaenidae		sp.3		X	P
Insecta	Coleoptera	Scydmaenidae		sp.4		X	A
Insecta	Coleoptera	Staphylinidae	Pselaphinae	sp.1		X	P
Insecta	Coleoptera	Staphylinidae	Pselaphinae	sp.2	X	X	E/P
Insecta	Coleoptera	Staphylinidae	Pselaphinae	sp.3	X	X	P/A
Insecta	Coleoptera	Staphylinidae	Pselaphinae	sp.13	X		P
Insecta	Coleoptera	Staphylinidae		sp.4	X	X	E/P
Insecta	Diptera	Chironomidae		sp.	X		A
Insecta	Diptera	Conopidae	*Stylogaster*	sp.	X		P
Insecta	Diptera	Drosophilidae	*Drosophila*	*eleonorae*	X		A
Insecta	Diptera	Psychodidae	Phlebotominae	sp.	X	X	E/P/A
Insecta	Diptera	Psychodidae	*Telmatoscopus*	sp.	X		E/P
Insecta	Diptera	Sciaridae		sp.		X	P/A
Insecta	Diptera	Tipulidae	Tipulinae	sp.	X	X	E/P
Insecta	Ephemeroptera	Leptophlebiidae		jovem		X	P
Insecta	Heteroptera	Cydnidae		sp.	X	X	P
Insecta	Heteroptera	Dipsocoroidea		sp.	X	X	P/A
Insecta	Heteroptera	Reduviidae	Emesinae	sp.		1	P
Insecta	Heteroptera	Reduviidae	*Panstrongylus*	jovem	1		A
Insecta	Heteroptera	Veliidae	*Paravelia*	sp.	X	X	E/P
Insecta	Homoptera	Cixiidae		jovem	X	X	E/P/A
Insecta	Hymenoptera	Diapriidae		sp.1		X	A
Insecta	Hymenoptera	Formicidae	*Camponotus*	sp.2	X	X	A
Insecta	Hymenoptera	Formicidae	*Crematogaster*	sp.1		X	P
Insecta	Hymenoptera	Formicidae	*Eurhopalothrix*	sp.1		X	A
Insecta	Hymenoptera	Formicidae	*Gnamptogenys*	sp.2		X	A
Insecta	Hymenoptera	Formicidae	*Hypoponera*	sp.1	X	X	A
Insecta	Hymenoptera	Formicidae	Myrmicinae	sp.1	X	X	P/A
Insecta	Hymenoptera	Formicidae	*Pachycondyla*	sp.1		X	E/P
Insecta	Hymenoptera	Formicidae	*Pachycondyla*	sp.2	X	X	E/P/A
Insecta	Hymenoptera	Formicidae	*Pheidole*	sp.1		X	E/P/A
Insecta	Hymenoptera	Formicidae	*Pheidole*	sp.2		X	E/P
Insecta	Hymenoptera	Formicidae	*Pheidole*	sp.3	X	X	P/A
Insecta	Hymenoptera	Formicidae	*Solenopsis*	sp.1	X	X	P/A
Insecta	Hymenoptera	Formicidae	*Trachymyrmex*	sp.1		X	P
Insecta	Isoptera	Termitidae	*Embiratermes*	sp.		X	E/P
Insecta	Isoptera	Termitidae	*Nasutitermes*	sp.	X		P/A
Insecta	Lepidoptera			jovem	X	X	P/A
Insecta	Orthoptera	Phalangopsidae	*Paraclodes*	sp.	60		E/P/A
Insecta	Orthoptera	Phalangopsidae	*Phalangopsis*	sp.	1	252	E/P/A
Insecta	Thysanura	Nicoletiidae		sp.1	X	X	P
Gastropoda	Pulmonata	Subulinidae		sp.2	X	X	E/P/A
Gastropoda	Pulmonata	Subulinidae		sp.4	X	X	A
Gastropoda	Pulmonata	Systrophiidae	*Happia*	sp.	X	X	P/A
Malacostraca	Decapoda			sp.	1		A
Malacostraca	Isopoda	Philosciidae		sp.1	X	X	P/A
Malacostraca	Isopoda	Scleropactidae	*Circoniscus*	*buckupi*	X		A

Malacostraca	Isopoda	Scleropactidae	*Circoniscus*	sp.2	X		A
Malacostraca	Isopoda	Scleropactidae		sp.1		X	A
Amphibia	Anura	Leptodactylidae	*Leptodactylus*	cf. *vastus*		1	E
Amphibia	Anura	Strabomantidae	*Pristimantis*	cf. *fenestratus*	1	1	P
Mammalia	Chiroptera	Phyllostomidae	*Carollia*	*perspicillata*	50		P/A
Mammalia	Chiroptera	Phyllostomidae	*Glossophaga*	*soricina*	1	2	P/A
Mammalia	Chiroptera	Phyllostomidae	*Micronycteris*	sp.		4	P/A
